# Alkali Niobate Powder Synthesis Using an Emerging Microwave-Assisted Hydrothermal Method

**DOI:** 10.3390/ma15155410

**Published:** 2022-08-06

**Authors:** Cristina-Rodica Dumitrescu, Vasile-Adrian Surdu, Hermine Stroescu, Adrian-Ionut Nicoara, Ionela Andreea Neacsu, Roxana Trusca, Ecaterina Andronescu, Lucian Toma Ciocan

**Affiliations:** 1 Department of Science and Engineering of Oxide Materials and Nanomaterials, Faculty of Chemical Engineering and Biotechnologies, University Politehnica of Bucharest, 060042 Bucharest, Romania; 2 National Research Center for Micro and Nanomaterials, Faculty of Chemical Engineering and Biotechnologyies, University Politehnica of Bucharest, 060042 Bucharest, Romania; 3 Institute of Physical Chemistry “Ilie Murgulescu”, Romanian Academy, 202 Splaiul Independentei, 060021 Bucharest, Romania; 4 National Research Center for Food Safety, University Politehnica of Bucharest, 060042 Bucharest, Romania; 5 Academy of Romanian Scientists, Splaiul Independentei Street No. 54, 011061 Bucharest, Romania; 6 Prosthetics Technology and Dental Materials Department, Carol Davila University of Medicine and Pharmacy, 020022 Bucharest, Romania

**Keywords:** alkali niobate powders, hydrothermal microwave-assisted synthesis, lead-free piezoelectric powders

## Abstract

For more than five decades, alkali niobate-based materials (K*_x_*Na_1−*x*_NbO_3_) have been one of the most promising lead-free piezoelectric materials researched to be used in electronics, photocatalysis, energy storage/conversion and medical applications, due to their important health and environmentally friendly nature. In this paper, our strategy was to synthetize the nearest reproductible composition to K*_x_*Na_1−*x*_NbO_3_ (KNN) with *x* = 0.5, placed at the limit of the morphotropic phase boundary (MPB) with the presence of both polymorphic phases, orthorhombic and tetragonal. The wet synthesis route was chosen to make the mix crystal powders, starting with the suspension preparation of Nb_2_O_5_ powder and KOH and NaOH alkaline solutions. Hydrothermal microwave-assisted maturation (HTMW), following the parameter variation *T* = 200–250 °C, *p* = 47–60 bar and dwelling time of 30–90 min, was performed. All powders therefore synthesized were entirely K*_x_*N_1−*x*_NbO_3_ solid solutions with *x* = 0.06–0.69, and the compositional, elemental, structural and morphological characterization highlighted polycrystalline particle assemblage with cubic and prismatic morphology, with sizes between 0.28 nm and 2.95 μm and polymorphic O-T phase coexistence, and a *d*_33_ piezoelectric constant under 1 pC/N of the compacted unsintered and unpoled discs were found.

## 1. Introduction

For several decades, the drastic reduction in natural fuel reserves led to the global energy crisis and renewable energy sources prompted researchers to explore alternative technologies. Therefore, the applications of piezoelectric energy harvesting in the top fields including smart systems, electromagnetic radiation, microfluidics, biomedicals, wearable and implantable electronics and tissue regeneration have been studied [1,2,3]. Sodium and potassium niobate K*_x_*Na_1−*x*_NbO_3_ (KNN)-based ceramics seem to be the most interesting piezoceramics to replace textured lead-based ones, the standard thanks to their highest piezoelectric features but causing an environmentally negative impact and human health unsafe behavior in use [4,5,6,7,8]. Ceramics based on KNN are secure from an environment point of view and show a promising combination of electro-mechanical properties and thermal stability [9,10,11,12,13,14]. However, the processing and sintering of such high-quality ceramics is still a challenge [15]. Bio-piezoelectric materials have attracted interdisciplinary research interest due to recent insights on the impact of piezoelectricity on biological systems and applications [16,17]. The piezoelectric effect observed at collagen physiological mineralization during new bone formation has leaded to the research of bio-piezo materials for tissue engineering able to improve osteopromotive and antibacterial effects [18,19,20,21]. The low piezoelectric effect (*d*_14_~0.2–2 pC/N) of such biopolymers (collagen, chitosan, etc.) is encountered in physiological mechanisms of bone retrieval [22]. Recent research leads to the conclusion that KNN-based ceramics could be designed to have a high piezoelectric coefficient (*d*_33_) and good temperature stability by the engineering of the composition at the Morphotropic Phase Boundary (MPB) [23,24,25,26,27,28]. Suitable KNN compositions located at the rhombohedral–tetragonal or rhombohedral–orthorhombic–tetragonal phase boundary were an effective choice to obtain an ultra-high *d*_33_ value. The piezoelectric coefficient can be tuned by size-effects in the submicrometer range and by using proper dopants, inducing electrically enhanced diffused polymorphic phase transition (EED-PPT) [28,29,30,31]. Moreover, shifting the phase-transition temperatures of KNN materials by doping or the formation of pseudo-binary solid solutions enhances the piezoelectric performance [32]. Classical synthesis approach involves advanced homogenization of reagents for long periods of time in waterproof conditions, multiple-step calcination at high temperatures, which causes undesirable phenomena such as the volatilization of alkalis with deviation from KNN stoichiometry and an excessive increase in particle size [33,34,35,36]. Pure KNN powders are difficult to sinter by the classical method, requiring high temperatures and long dwelling time, that may cause volatilization and increased porosity as well as the conservation of the paraelectric cubic symmetry phase at room temperature [32,37]. The synthesis at high temperatures also leads to an increase in particle size in the micrometer range [38,39]. The related physical mechanisms to obtain exactly the suitable composition of K_0.5_Na_0.5_NbO_3_ solid solution at MPB need further exploration, even if high piezoelectricity could be achieved only with satisfactory traceability [27,40,41]. Therefore, the use of a wet hydrothermal (HT) or solvothermal (ST) synthesis method for obtaining a nano- or micro-structured powder of K_0.5_Na_0.5_NbO_3_ seems to be the best choice [42,43,44,45]. Moreover, the HT/ST method allows better temperature/pressure control, together with a proper precursor mixture, leading to a predictable particle morphology [46]. Using HT synthesis, starting with Nb foil, at 200 °C for 4 h, sodium niobate nanowires have been successfully synthetized [47]. Single crystalline KNN particles have been synthetized by HT method at 220 °C with a soaking time of 24 h [48]. For an efficient heating, microwave system can be used assisting the HT method or for sintering step [43]. Microwave-assisted hydrothermal synthesis method combines the advantages of high-pressure wet technology of hydrothermal (HT) with microwave (MW) heating system on a previously prepared suspension, which leads to higher compositional and submicron-sized particles homogeneity [10,46]. Microwave radiation was volumetrically absorbed leading to the conversion of electromagnetic energy into heat in the volume of irradiated material due to intermolecular friction and, as a result, diffusion processes were enhanced, energy consumption was reduced, heating rates were very rapid and synthesis times were considerably reduced [43]. Moreover, thanks to MW energy, molecules from the aqueous suspension polarize and will easily couple with rapid electric field reversal [49]. MW can be absorbed by materials either by polarization or by ionic conduction processes [50]. The material absorption of MW energy depends on the incident MW frequency, the dielectric constant of the material, the dielectric loss of the material and the distribution of the electric field inside the material, that make possible the better technological parameters control and a proper induced kinetic [49,51,52,53,54]. New research showed high ferroelectric performances of pure KNN ceramics sintered by Spark Plasma Sintering technique, starting with K_0.5_Na_0.5_NbO_3_ submicron powder [55,56].

The aims of this study were to define the technological conditions for a predictable K*_x_*Na_1−*x*_NbO_3_ powder synthesis with *x*~0.5, submicron-sized particles, with the co-existence of orthorhombic (O)–tetragonal (T) crystal symmetry phases at room temperature, by the means of the hydrothermal microwave-assisted (HTMW) method. The desired composition of the KNN solid solution was achieved after a short dwelling time and low temperature of HTMW treatment, with minimum energy consumption. For this reason, we investigated the influence of the selected technological parameters (precursors solution concentration, temperature and duration) over the sodium and potassium content in KNN, powder that could be utilized as raw material for piezoelectric ceramics or the new generation of smart bio-composite fabrication.

## 2. Materials and Methods

### 2.1. Regent Suspension Preparation and Hydrothermal Microwave-Assisted (HTMW) Maturation

Flakes of KOH (Reagent purity, Sigma-Aldrich, St. Louis, MI, USA) and NaOH (≥98%, Honeywell) in different amounts according to the recipes (Table S1 Supplementary data), 1.5 g Nb_2_O_5_ powder (99.9% Fulka AG, Buchs SG, Switzerland) was used to obtain the reagent suspensions. The alkaline solutions were prepared by mixing the excess at required amounts of KOH and NaOH in 30 mL of distilled water with magnetic homogenization maintained until complete solubilization, then the amount of 1.5 g Nb_2_O_5_ was added, continuing the homogenization for 30 min. The aim was to obtain suspensions by varying both the concentrations of the alkaline precursor solutions KOH + NaOH = 10M and 16M, and KOH/NaOH molarity ratio variation within the limits of 1:1, 7:3, 3:2, 4:1. All suspensions were obtained with alkaline molarity in excess in order to increase the reaction kinetic with niobium penta-oxide powder, whose structure is chemically very stable [42]. The prepared samples were labeled according to the molarity of each alkaline precursor, temperature of the reaction and soaking time as shown in Table S1, supplementary data (for example for the sample obtained by using 8M KOH, 8M NaOH, a reaction temperature of 200 °C and a dwelling time of 30 min the label is K8N8_200_30). The four different suspensions K8N8, K8N2, K7N3 and K6N4 were poured into Teflon containers with 60% filling ratio for HTMW synthesis.

The variation in the input HTMW technological parameter progress applied (time–temperature–pressure) was observed. The entry parameters were settled: pressure at 40 bars, heating ramp 25–200 °C or 25–250 °C: 13.34 °C/min, a cooling rate of 10.66 °C/min and variable dwelling time 30, 60 and 90 min. The pressure in the system varied from 40–47 bar for 200 °C cycle and 40–60 bars for 250 °C, the microwave energy supplied to the system for heating to 200 °C or 250 °C reached a maximum of 1.35 kW and 1.5 kW, respectively.

### 2.2. Characterization Methods

X-ray powder diffraction (XRPD) was performed using PANalytical Empyrean Diffractometer (Malvern PANalytical, Almelo, Netherlands), operating in a Bragg–Brentano configuration with Cu-Kα (*λ* = 1.5406 Å) with a working voltage and current of 45 kV and 40 mA, respectively. The spectra were recorded between 10° < 2*θ* < 80° with a counting time per step of 255 s and a step size of 0.02°.

In order to calculate the average crystallites size of K*_x_*Na_1−*x*_NbO_3_ powders, the Rietveld method was applied, based on all profiles’ X-ray diffraction peaks in the pattern, using HighScorePlus 3.0.e software and pseudo-Voigt function for profile refinement procedure [57].

The Goldschmidt tolerance factor has been calculated (Equation (2)) for perovskite structure ABO_3_ [58], by using a modified Equation (2) for a solid solution K*_x_*Na*_(_*_1−*x)*_NbO_3_ adapted from Equation (1) [59].

*t* = (*R_A_* + *R_O_*)/(√*2*(*R_B_* + *R_O_*)) (1)

*t* = (*xR_K_* + (*1* − *x*)*R_Na_* + *R_O_*)/(√*2*(*R_Nb_* + *R_O_*)) (2)

where *t* = Goldschmidt tolerance factor; *R_A_, R_B_* = ion radius of A or B position in ABO_3_ perovskite lattice; *R_O_* = ion radius of oxygen; *R_K_, R_Na_, R_Nb_* = ion radius of K, Na and Nb; and *x* = molar fraction in K*_x_*Na_1−*x*_NbO_3_

A Quanta Inspect F scanning electron microscope (SEM) (Thermo Fisher, Eindhoven, The Netherland), equipped with field electron emission gun (FEG) and an EDS detector (Energy Dispersive Spectroscopy) was used. The technical parameters were an acceleration voltage of 30 KV and a point-to-point resolution of 1.2 nm. The histograms of the samples were obtained from the statistical processing of images using the ImageJ software.

A TECNAI F 30G2 SWIN transmission electron microscope (TEM) (Thermo Fisher, Eindhoven, The Netherland) was used, with 300 KV acceleration transmission with Schottky electron emission, HRTEM point and line resolution 2 Å and 1.02 Å, respectively, 60x-1Mx magnification range, minimum diffraction angle ±12°, equipped with an EDS probe.

Thermo Scientific™ ARL ‘PERFORM’X Sequential X-ray Fluorescence Spectrometer (XRF) was used for the elemental composition of the powders, which works under pressure in the He atmosphere, the acquisition is made according to the Thermo Scientific™ UniQuant soft, with a non-standardized method.

Raman spectra were measured in the normal atmosphere (air), at room temperature, in the range of 0–1000 cm^−1^, using the green line of an Ar laser (*λ* = 514 nm) on a Horiba JobinYvon LabRam HR spectrometer with a power below 50 mW/sample. Collected spectra were analyzed using Witec Control Plus Software, and Raman mode positions were fit assuming a Lorentz peak shape.

The piezoelectric coefficients *d*_33_ were measured for the unsintered and unpoled KNNss disks, using a PiezoMeter System PM300 from Piezotest Pte. Ltd. (Singapore). The top and the backside of the samples were covered with silver paste and the measurements were calculated using a frequency range between 30 and 110 Hz, using a static force of 10 N and a dynamic force of 0.05 N, without sample poling. The discs of *Φ* = 6 mm were prepared using unsintered powders and 0.1 wt. % APV, after pressing at 550 N/mm^2^ in a metallic mold.

## 3. Results and Discussion

The maximum temperature of the HTMW trials varied up to 250 °C, a higher temperature being prevented by equipment limitation, and a lower temperature than 200 °C involving the risk of incomplete reaction with remanent secondary phases [60] and single orthorhombic phase formation [42].

### 3.1. X-ray Fluorescence Spectra

The influence of technological parameters of the HTMW method (aqueous solution concentration, temperature and entire treatment time) on the KNN compositions (molar fraction x) was achieved using XRF, XRD, Raman spectra analysis and semi-quantitative EDS assessment.

For XRF result validation, the following assumptions were imposed: the entire quantity of each powder synthesized has a certain Na or K determined content, bounded into one phase structure of KNN. The estimated formula for each KNN sample is shown in Table 1 and Table S2 (Supplementary data).

The interpretation of the EDS spectra on five areas of each specimen highlighted the elements present on the scanned samples: Nb, Na, K, O (Table 1). Additionally, Table 1 summarizes the estimated formulas of KNN, obtained by calculation from the quantitative XRF analysis of around 3 g of every synthetized sample (Table S2 Supplementary data) and EDS spectra. From these data, it is observed that the fifteen different samples also generate fifteen KNN compositions and only certain samples approached the targeted K_0.5_Na_0.5_NbO_3_ (K8N8_200_90, K7N3_200_30, K8N2_250_30).

**Figure 1:**
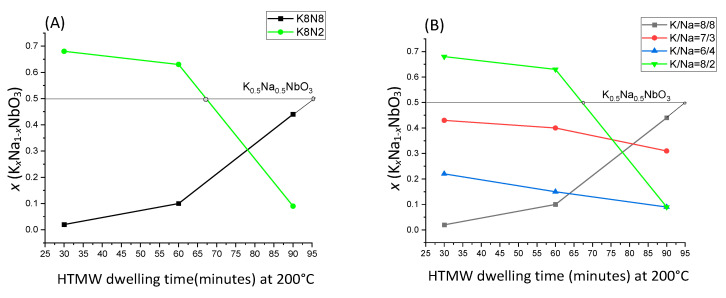
Solid solution K*_x_*Na_1−*x*_NbO_3_ molar fraction *x* variation depending on HTMW dwelling time (30–90 min) at maximum temperature 200 °C, for different alkaline precursor mix molar rates. (**A**) K8N8 sample vs. K8N2 (KOH-8M); (**B**) samples K7N3, K6N4 and K8N2 (KOH + NaOH = 10M).

**Figure 2:**
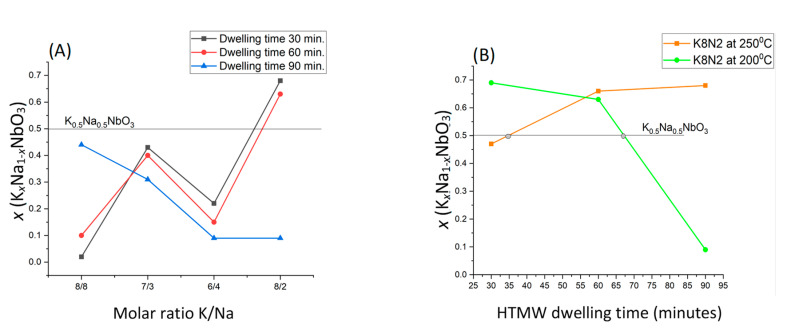
(**A**) Molar fraction *x* (K*_x_*Na_1−*x*_NbO_3_) variation depending on alkaline precursor molar rate for the three series of HTMW dwelling time (30, 60 and 90 min) at maximum temperature 200 °C; (**B**) Solid solution K*_x_*Na_1−*x*_NbO_3_ molar fraction *x* variation for sample K8N2 (K/Na = 8M/2M) depending on the HTMW temperature 200 °C and 250 °C at the three tested dwelling times (30, 60 and 90 min).

Assuming the XRF findings, at constant temperature (200 °C), the influence of different precursor concentration effects over the *x* fraction of KNN, at different dwelling times, reveals two opposite behaviors (Figure 1A,B): in the case of precursor mix (KOH + NaOH) = 10M, the K^+^ content of KNN decreased with increasing dwelling time, and (KOH + NaOH) = 16M, Na^+^ content decreased over time. In Figure 1A, it is observed that at the same KOH concentration (8M), at short HTMW treatment (30 min), a solid solution rich in K (*x* = 0.69) is obtained for K8N2, and in contrast, for K8N8, a low *x* value (*x* = 0.06) of KNN is obtained. For the sample K8N2_200_30, the KOH concentration (8M) is four times higher than NaOH (2M) one, and potassium is also more reactive than sodium; therefore, after 30 min of treatment, K_0.69_Na_0.31_NbO_3_ is expected to be the composition. The K^+^ content slowly decreased after 60 min (*x* = 0.63) because Na^+^ started to substitute it due to its smaller ion radius (1.39 Å for a number of coordination of 12); thus, after 90 min of treatment, the KNN became Na rich (1 − *x* = 0.91). For the second sample (K8N8), where Na is equimolar with K, at 30 min, the higher diffusion mobility of Na^+^ dictated the content of KNN (K_0.06_Na_0.94_NbO_3_). The process of Na substitution by K from perovskite structure continue in time, due to its higher reactivity, and K_0.44_Na_0.56_NbO_3_ is obtained after 90 min. In Figure 2B, the same formation–dissolution mechanism is observed for the samples K7N3 and K6N4 as per K8N2, but the ratio KOH/NaOH ~2 for these two samples induced moderate changes in KNN compositions in time.

The targeted KNN (K_0.5_Na_0.5_NbO_3_), at the same KOH/NaOH ratio and temperature (200 °C), could be reached by the variation in dwelling time for the series of samples K8N8 after 90 min of HTMW treatment (*x* = 0.44), for K7N3 after 30 min (*x* = 0.43) and apparently cannot rich a higher molar fraction *x* over 0.43, for the samples in series K6N4, the highest K content was 22 molar % at 30 min (Figure 2A).

In the series of samples K8N4, the maximum temperatures 200 °C and 250 °C of HTMW treatment were compared as the effect of *x* variation in the resulting KNN (Figure 2B). After the short HTMW cycle, the closer *x* to 0.5 (*x* = 0.47) was obtained at 250 °C (K8N2_250_30), but after data extrapolation (Figure 2B), the composition K_0.5_Na_0.5_NbO_3_ the would be expected at 35 min of treatment. The same reasoning could be considered at composition K8N2 at 200 °C if the HTMW dwelling time would be 67 min instead 60 min.

### 3.2. X-ray Powder Diffraction (XRPD) and Rietveld Structure Refinement

**Figure 3:**
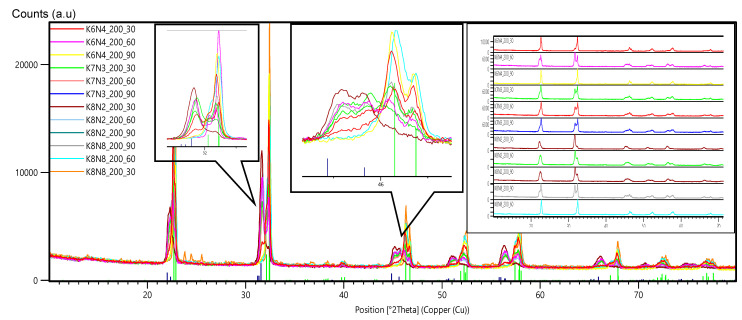
X-ray powder diffraction plot for the samples’ HTMW synthetized at 200 °C, 30–90 min, compared with orthorhombic NaNbO_3_ XRPD pattern (green, PDF 04-012-8146) and orthorhombic KNbO_3_ XRPD pattern (black, PDF 04-014-4292), graphics displayed successively and overlapped plots with upper insert detailed sector 2*θ* = 31–33° and 44–46°, respectively.

Crystalline phases identified by X-ray powder diffraction in the 15 synthetized samples highlight the formation of fifteen distinct solid solutions (K*_x_*Na_1−*x*_NbO_3_), which confirms XRF findings characterized by different K and Na content (*x* = 0.06 − 0.69), as could be seen in Figure 3. The off-centering of Nb inside the octahedra, rotations, tilting and distortions of the corner-connected NbO_6_ octahedra determine the Nb-O-Nb bond angles and Nb-O bond length deviation and lead to the appearance of abnormalities in perovskite lattice characteristics, highlighted by X-ray diffraction profile deformations and difficulties in XRD pattern matching [61]. Moreover, the presence of polymorphous phases (orthorhombic–tetragonal or orthorhombic–tetragonal–monoclinic) onto the analyzed powders gives rise to even more interpretations in comparison with the Powder Diffraction Files (PDF) in the database (ICDD) that referred to a synthesized single phase powder [61]. The relative intensity differences could be also attributed to smaller or larger particle size, as well as the broadening of some reflection peaks [62]. However, XRD analysis did not allow to identify each of the fifteen K*_x_*Na_1−*x*_NbO_3_ composition, with dissimilar molar fraction *x* for more reasons: the ICDD-PDF database has insufficiently available patterns for such a wide range of KNN compositions, with more unit cell symmetry coexistence, different from nano- and submicronic particles [26]. Obviously, all compositions synthetized by HTMW method at 200 °C for 30–90 min, fit between XRD orthorhombic patterns of NaNbO_3_ (green, PDF 04-012-8146) and orthorhombic KNbO_3_ (black, PDF 04-014-4292), the two limits of K*_x_*Na_1−*x*_NbO_3_ solid solution (*x* = 0.06 − 0.69). No secondary phases as unreacted KOH, NaOH and Nb_2_O_5_ have been found. For the samples K8N2_200_30, K8N2_200_60, K7N3_200_30 and K8N8_200_90, we observed the tendency to record the highest intensity of diffraction peaks at 2*θ* ~ 31^0^ close to the black-colored maximum intensity (100%) of orthorhombic KNbO_3_ (2*θ* = 31.5°), which can be associated with a higher K content of KNN (Figure 3, inserts). On the contrary, the samples K8N8_200_30, K8N8_200_60, K8N2_200_90, K6N4_200_90, K6N4_200_60 and even K6N4_200_30 (Figure 3, inserts) placed the maximum diffraction peaks (100%) over the angle 2*θ* = 32°, which could demonstrate their higher content of Na, similar to in orthorhombic NaNbO_3_ (2*θ* = 32.4°).

The split peaks at 2*θ* around 22° reflected by crystalline plane (110)/(001), as well as at 32° for (020/(200)/(111) and 2*θ*~45° (200/002) O, (002)T, (200)T are characteristic of KNN patterns and also mark the coexistence of O/T crystallization symmetry at the room temperature. Considering that K_0.5_Na_0.5_NbO_3_ have O symmetry and it is thermodynamically stable below 200 °C, and T symmetry occurs above this temperature, the formation of KNN solid solution with both symmetries after hydrothermal treatment between 200 and 250 °C is expected [63].

**Figure 4:**
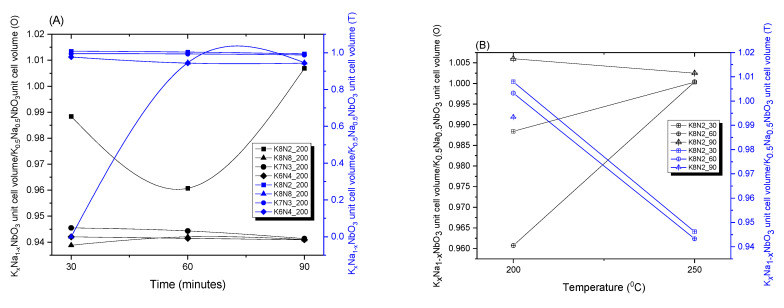
Unit cell relative volume variation depends on dwelling time (**A**) and temperature (**B**) of HTMW treatment for series of samples: K8N8, K8N2, K7N3 and K6N4.

Rietveld structural refinement for all analyzed samples was performed using a tetragonal symmetry (T, P4*mm*, PDF 04-017-0217), orthorhombic symmetry (O, A*mm*2, PDF 04-017-0216) and monoclinic (M, P*m*6, PDF 00-061-0315) of K_0.5_Na_0.5_NbO_3_ diffraction patterns. The results of the refinement highlighted the similarity of unit cell volume (parameters) of analyzed samples with the patterns. Diffraction peak shapes were fitted assuming a Lorentz function. The calculated lattice parameters, phase content and crystallinity can be observed in Figure 4A,B and Tables S3–S5 (Supplementary data).

For all samples in T phase, the ratios V_sample_ (T)/V_K0.5Na0.5 NbO3_ (T) were almost ~1, no matter the dwelling time (Figure 4A, blue chart), proving the similarity of unit cell volume of samples and K_0.5_Na_0.5_NbO_3_ pattern. In case of O phase, a low ratio V_sample_ (O)/V_K0.5Na0.5 NbO3_ (O) << 1 was observed for all samples (Figure 4A, black chart). Greater distortions caused by a higher (+) or (−) octahedra tilting manifested in a larger O unit cell volume of samples compared with the O unit cell of K_0.5_Na_0.5_NbO_3_ pattern (PDF 04-017-0216 [64]. The exception manifested at the sample K8N2_200, for both 30 and 60 min dwelling times. At these samples, the unit cell volume rates increase in T phase from 0.94 (30 min) to 1.01 (60 min) and decrease in O phase from 0.99 (30 min) to 0.96 (60 min) [63].

According to recent research, the variation in the tolerance factor *t* was related with different kinds of perovskite unit cell distortions [65]. Octahedral NbO_6_ tilts may be associated with the Goldschmidt’s tolerance factor *t*, used to evaluate perovskite geometrical stability and distortion at the atomic level [59].

**Figure 5:**
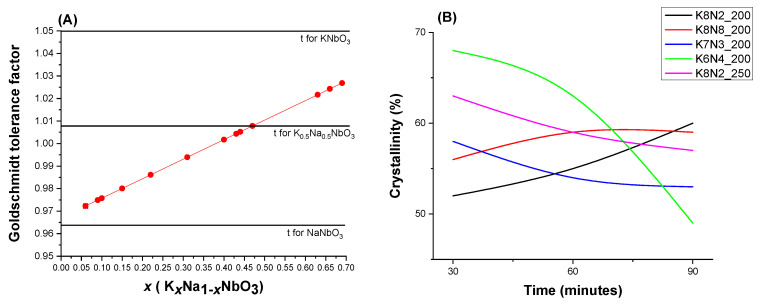
(**A**) Variation in Goldschmidt tolerance factor *t* up to molar fraction *x* from K*_x_*Na_1−*x*_NbO_3_. (**B**) Crystallinity degree for all series of powders K8N2_200, K8N2_200, K8N2_250, K7N3_200, K6N4_200 HTMW synthetized for 30–90 min.

Figure 5A and Table S6 (Supplementary data) show the variation in the tolerance factor *t* with the molar fraction. As observed, the values of *t* = 1.0537 (*t* ≥ 1) for KNbO_3_ perovskite lattice were explained by a high stability of NbO_6_ octahedra against tilting, conversely for NaNbO_3_ with *t* < 1 (0.964) numerous distortions and tilts in variable symmetry phases were reported [58,59]. The lattice of K*_x_*Na_1−*x*_NbO_3_ forms stable solid solutions exhibiting excellent piezoelectric properties in the vicinity of *x* = 0.5 [65], where the calculated factor *t* is 1.007. As observed, all samples K*_x_*Na_1−*x*_NbO_3_ show better atom packing into a perovskite unit cell; therefore *t* values are closer to 1, comparing to the two limit compounds of KNN (KNbO_3_ and NaNbO_3_). The closest *t* to 1 (cubic perovskite symmetry) is calculated for the samples K7N3_200_30 (t = 1.001), K8N8_200_90 (t = 1.002), K8N2_250_30 (t = 1.004). Additionally, it is observed that the higher the value of *t* is, the higher the K content in KNN. In contrast, the lower the *t*, the higher the Na content in K*_x_*Na_1−*x*_NbO_3_. Following the model of KNbO_3_ perovskite structure (*t* = 1.05), the samples K8N2_200_30 or K8N2_250_90 with *t* = 1.021, close to 1.05, will have the highest K^+^ content (*x* = 0.68 and 0.69, respectively), even higher than sample K8N2_200_60 or K8N2_250_60 with lower *t* values *(t* = 1.018) and *x* = 0.63 and 0.66, respectively. These observations confirm the XRF findings.

The crystallinity degree has been determined for all four series K8N8, K8N2, K7N3 and K6N4 at 200 °C and 250 °C, dwelling time 30–90 min (Figure 5B, Tables S3–S5 Supplementary data). The powder crystallinity decreases with increasing dwelling time for all specimens, but in series K6N4_200 with a steep slope and in a moderate way for K7N3 at 200 °C. Such behavior could be influenced by the high energy provided by microwave heating source, which induces an improved dissolution process that progresses further in time.

**Figure 6:**
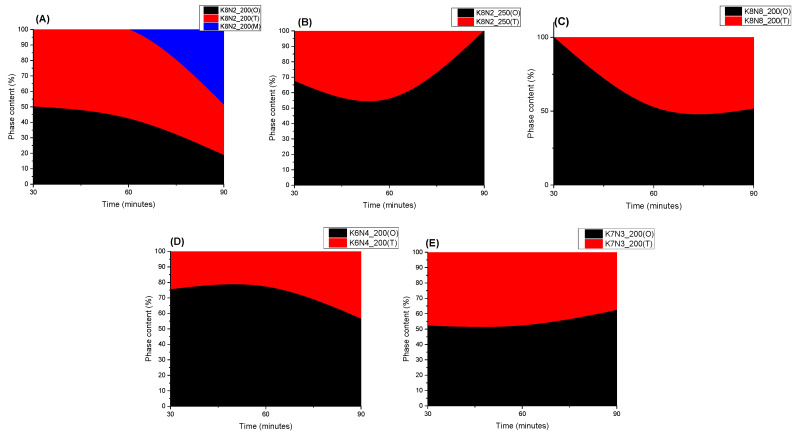
Monoclinic, orthorhombic and tetragonal phase content of HTMW-synthetized powders in series K8N2_200 (**A**), K8N2_250 (**B**), K8N8_200 (**C**), K6N4_200 (**D**) and K7N3_200 (**E**), for 30–90 min.

The polymorphic phases identified after Rietveld refining are represented by O in a majority of the synthesized powders, both at 200 °C and 250 °C, no matter the dwelling time. The samples with high Na content crystals were developed on O sodium niobate symmetry and tend to contain a high proportion of this phase (K6N4_200_30 with 75% O, K6N4_200_60 with 77% O). K8N2 series samples is an exception which possess the third monoclinic phase that appears at 200 °C and 90 min (Figure 6, Tables S3–S5 Supplementary data). Apparently, the higher temperature 250 °C does not affect the proportion of O phase, which remains the major one. In correlation with *t* factor, as reported, a tolerance factor *t* = 1 raised for the highest unit cell symmetry was also observed for the samples K7N3_200_30 (*t* = 1.001), K8N8_200_90 (*t* = 1.002) and K8N2_250_30 (*t* = 1.004), showing an increased content of T phase at around 50% (Figure 6) [65].

**Figure 7:**
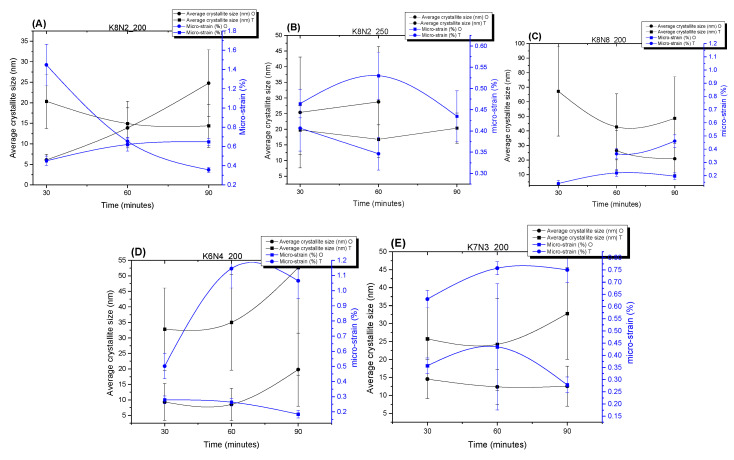
Average crystallite sizes and micro-strain variation in KNN particles’ orthorhombic and tetragonal symmetry, crystallized depending on the HTMW dwelling time at 200 °C and 250 °C, for the series of samples K8N2_200 (**A**), K8N2_250 (**B**), K8N8_200 (**C**), K6N4_200 (**D**) and K7N3_200 (**E**).

In connection with unit cell volumes and tolerance factors, a higher structure micro-strain creates the tendency of symmetry decrease for relaxation and better phase stability [64]. As expected, the larger the crystallite size, the lower the micro-strain is (Figure 7). As expected, lower values of micro-strain have been found at O phase, the average size of T crystallites being smaller than O ones, which induces a higher micro-strain in the particles of K8N2_200_30, K6N4_200_60 and K7N3_200_60 powders (Figure 7, Tables S3–S5, supplementary data).

### 3.3. Raman Spectra

**Figure 8:**
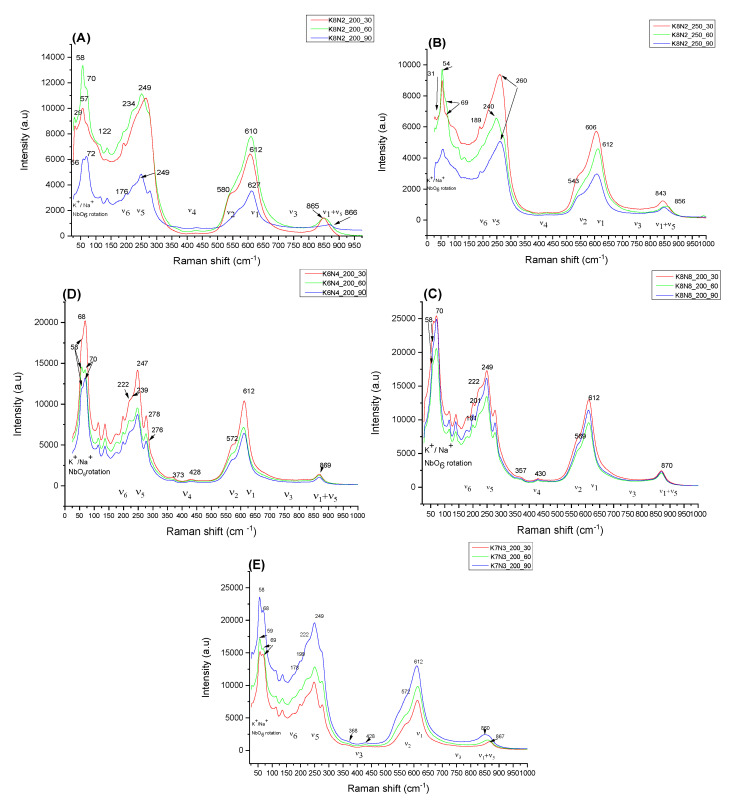
Comparative Raman spectra for the series of samples K8N2_200_30–K8N2_200_90 (**A**), K8N2_250_30–K8N2_250_90 (**B**), K8N8_200_30–K8N8_200_90 (**C**), K6N4N_200_30–K6N4N_200_90 (**D**) and K7N3_200_30-K7N3_200_90 (**E**).

Raman spectra obtained on the KNN solid solutions are shown in Figure 8A–E and are in good agreement with XRF and XRD results. The peaks in the low frequency range (0–150 cm^−1^) are attributed to the translation mode of K^+^ (28, 55 and 58 cm^−1^), Na^+^ (68–73 cm^−1^) and NbO_6_^7−^ octahedra at 113 and 136 cm^−1^ [36,66]. By looking at the intensities and the frequencies observed for the specific bands of K^+^ and Na^+^, one can have an understanding on the K^+^/Na^+^ content in the obtained solid solutions. Therefore, on the one hand, solid solutions with a higher content of K^+^ show higher intensities for the specific translation modes and a shift in the peaks in lower frequency modes (K8N2_200_30, K8N2_200_60, K8N2_250_60 and K8N2_250_90), and, on the other hand, solid solutions with higher Na^+^ content show specific modes with higher intensity (K6N4_200_90, K8N8_200_30, K8N8_200_60) in the 68–73 cm^−1^ range.

In the wide frequency range (150–900 cm^−1^), three active Raman modes (ν_1_A_1g_, ν_2_E_g_, ν_5_F_2g_) are associated with the symmetric octahedra of NbO_6_^7–^ bond vibrations. Moreover, inactive Raman modes (ν_3_F_1u_, ν_4_F_1u_, ν_6_F_2u_) can be detected with low intensities at 178, 199, 373, 428 cm^−1^ frequencies [36]. The shift in the Raman modes, as well as the profile of the peaks in the wide frequency range provide clues of the lattice symmetry specific to the analyzed samples. Therefore, the distortion of the O-Nb-O bond in the NbO_6_^7−^ octahedra, specific to tetragonal to orthorhombic transition, is evidenced by a shift in the stretching vibrational mode A_1g_(ν_1_) from 606, 610 or 612 cm^−1^_._ The transition is also marked by peak splitting and intensifying the shoulder at 569–572 cm^−1^ by vibrational stretching mode E_g_(υ_2_), more obvious for samples K6N4_200_30 (572 cm^−1^), K8N2_200_30 (580 cm^−1^), K8N2_250_30 (543 cm^−1^) and K8N2_250_90 (540 cm^−1^) [33]. Moreover, when the crystallization symmetry changes, the active bending mode F_2g_ (υ_5_) becomes more intense and shift to a higher frequency (850–870 cm^−1^), which is visible for all analyzed samples [66,67].

### 3.4. Scanning Electron Microscopy

**Figure 9:**
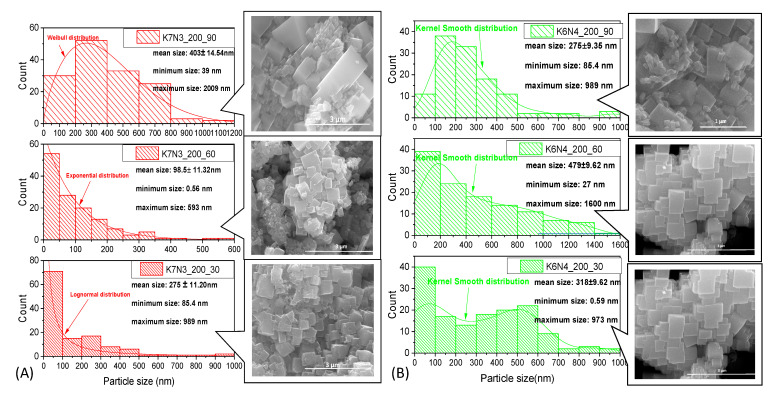

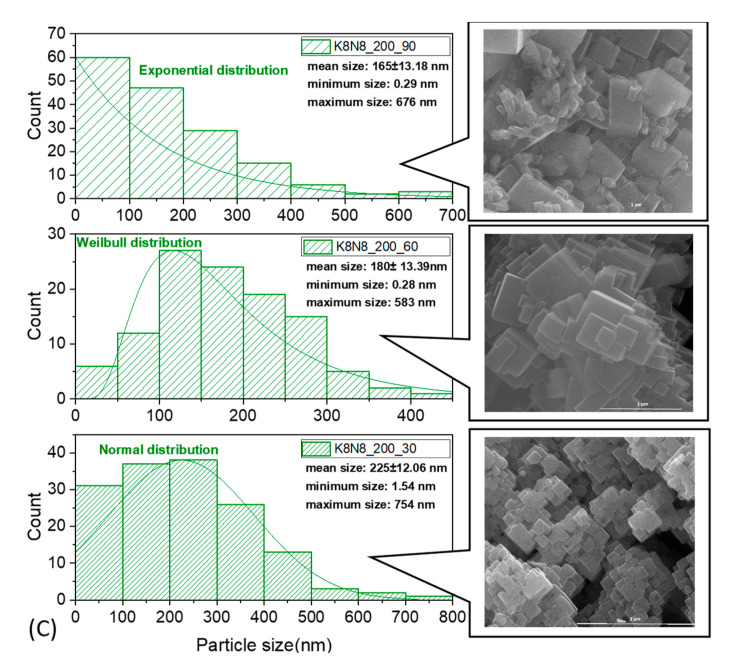
SEM images and particles size distribution for sample series K7N3 (**A**), K6N4 (**B**) and K8N8 (**C**), after 30–90 min of HTMW at 200 °C.

**Figure 10:**
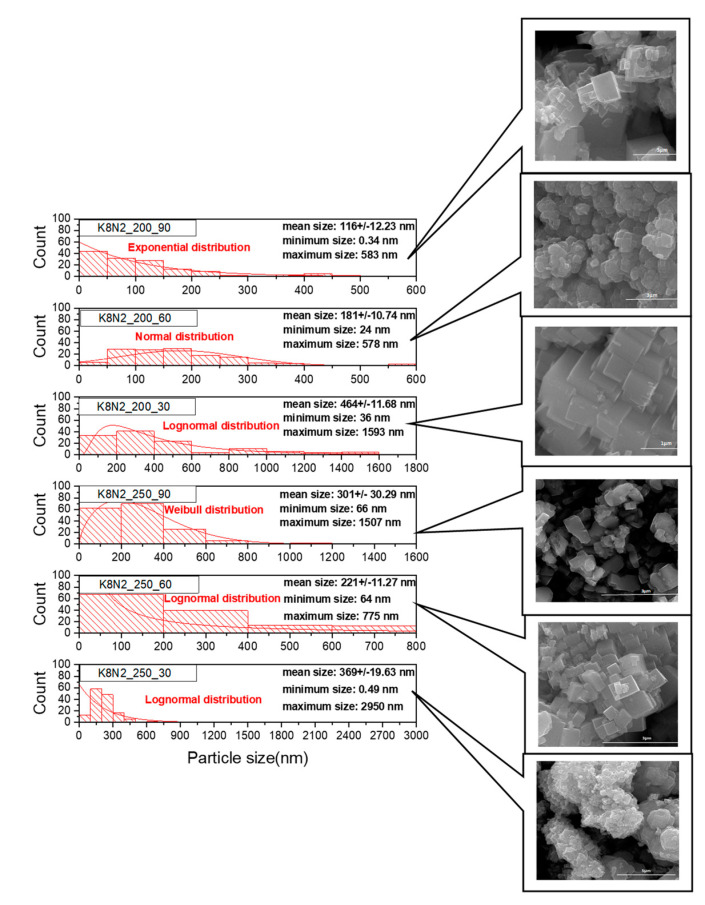
SEM images and particles size distribution for sample series K8N2 after 30–90 min of HTMW at 200 °C and 250 °C.

SEM images (Figure 9A–C and Figure 10 and Table S7, supplementary data) for all K*_x_*Na_1−*x*_NbO_3_ solid solutions show the presence of well-crystallized agglomerations with a prismatic and cuboid shape, a continue granulation with micrometric course limit below 1% of grains and more than 65% fine grains at nanometric sizes limit, with smooth (K7N3_200_60, Figure 9A) or rough square faces (K8N2_200_60, Figure 10), with straight (K6N4_200_30, Figure 9B) or slightly rounded edges and corners (K8N2_250_90, Figure 10). Right edges are well defined especially at shorter treatment times (30 min). As the dwelling time increases at 60 and 90 min, the corners of the prisms become rounded, caused by the dynamics of the competitive processes of dissolution/nucleation, agglomeration or splitting and crystal growth. Additionally, coalescence nano-sized crystal growth over large crystals is observed (K8N8_200_30, Figure 9C), oriented along planes with small Miller indices ((100), (010) and (001)) [36].

The microwave radiation has an accelerating effect of nucleation versus crystal growth, so powders obtained after a short time (30 min) generally have small sizes, such as the sample K7N3_200_30 which contains 70% particles under 100 nm and K/Na content ~1. The gradual increase in grain sizes with dwelling time (60 and 90 min) was not confirmed for all samples. The largest particle sizes are generally found in specimens with longer treatment durations (K6N4_200_60 by 1.6 μm, K7N3_200_90 by 1.209 μm) and at higher temperatures (K8N2_250_60 of 2.95 μm), which accelerates crystal growth compared to the nucleation process.

In the K8N2 series (Figure 10 and Table S7, supplementary data), the average grain sizes decrease with the decrease in the K content of K*_x_*Na_1−*x*_NbO_3_, caused by the higher ionic radius of K (1.64 Å), compared with Na^+^ (1.39 Å), which induces the formation of larger crystals and crystallites. Therefore, for sample K8N2_200_30 (*x* = 0.69), the average crystal size is 463.6 nm and it decreases at 116 nm in K8N2_200_30 (*x* = 0.09). Additionally, in the same series (K8N2), after temperature change from 200 to 250 °C, *x* values increase with dwelling time, sample K8N2_250_30 (*x* = 0.47) has the smallest crystal size (221.3 nm) compared with K8N2_250_90 (*x* = 0.68), which is 301 nm. The stresses occurred at the transition from the low symmetry phase (M or O) to higher symmetry (T) lead to the reduction in the elementary cell volume, crystallite sizes and morphotropy, and KNN particle size decreases.

**Figure 11:**
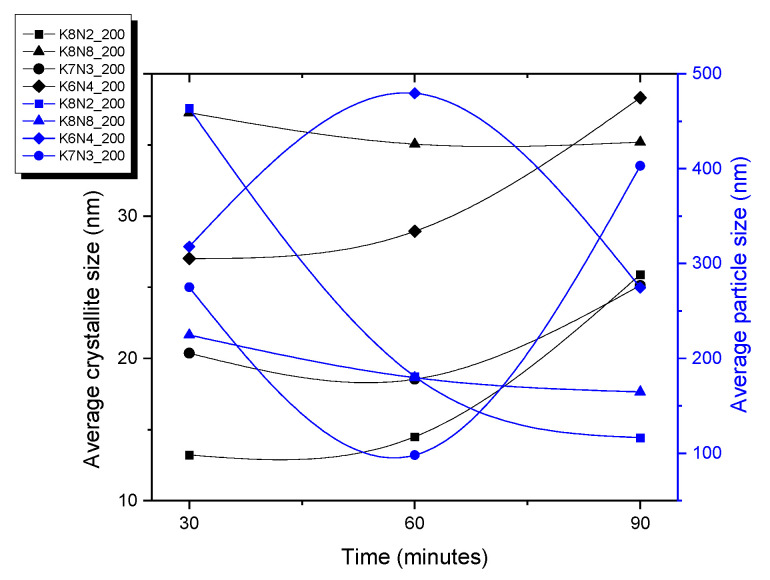
Average of crystallite size and particle size of all samples at 200 °C, depending on the HTMW dwelling time (30–90 min).

Two opposite behaviors of the samples were observed at 200 °C and 30–90 min dwelling time, up to precursor mix KOH + NaOH. For the first group, with KOH + NaOH = 10M, the crystallite size tends to increase with increasing time (K8N2, K7N3 and K6N4) (Figure 11 and Tables S3–S5, supplementary data). However, the proportional dependence on the crystallite, crystal size and time is not fully understood. For K8N8 series (KOH + NaOH = 16M) with a high content of orthorhombic phase, crystallite sizes (42–48 nm) are almost double compared with all other series of samples (<32 nm) (Figure 9C, Tables S5 and S7, supplementary data). The sample K8N8 synthetized at 30 min only contains O phase; therefore, larger crystallites create larger polycrystalline particles by assemblance. The important tendency to crystalize in O symmetry, with higher crystallite sizes, was reported for KNN with high Na content compared with those rich in K [42].

### 3.5. Transmission Electron Microscopy (TEM)

**Figure 12:**
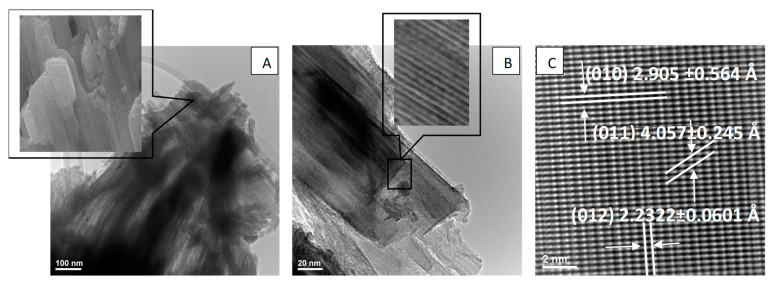
TEM (**A**,**B**) and HRTEM (**C**) images for particles of the sample K8N2_200_30.

**Figure 13:**
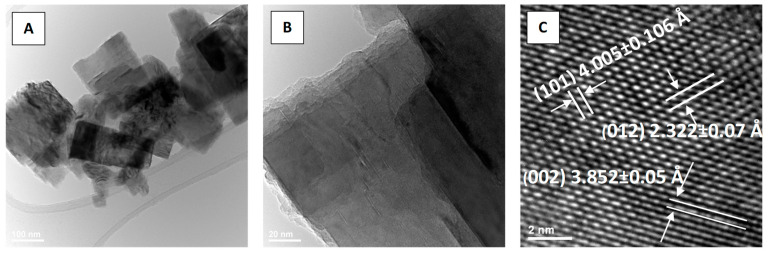
TEM images (**A**,**B**) and HR-TEM (**C**) image for particles of the K8N2_250_30 sample.

TEM images for the sample K8N2_200_30 (Figure 12A) reveal a particle morphology which resembles a bunch of ribbons oriented in the same direction. The B image of Figure 13 confirms the same overlapping assembly of the single crystal (left insert) with a thin sheet morphology with micrometric length, observed in the SEM image (100,000 X) in the right site insert. Thus, from the HRTEM images, the measurement (*n* = 50) of the interatomic distances (d-spacing) 3.941 Å, 5.089 Å and 5.608 Å for K8N2_200_30 sample (Figure 12C) was attributed to tetragonal symmetry, verifying the correspondence with the Miller index planes (100), (020) and (210) of K_0.5_Na_0.5_NbO_3_ tetragonal symmetry (P4*mm*).

For K8N2_250_30 sample, TEM images (Figure 13A–C) reveal the rectangular platelet crystal morphology with an average size of 182.7701 ± 8.5908 nm (*n* = 20), with evidence of crystallite orientation according to the same plane families. Accordingly, for sample K8N2_250_30, the inter-atomic distances measured in HRTEM images 2.322 Å, 3.852 Å and 4.005 Å correspond to the 2*θ* diffraction angles of 38.99°, 31.49° and 22.15° attributed to the orthorhombic symmetry, and the crystallographic planes (012), (002) and (101) attributed to orthorhombic symmetry K_0.5_Na_0.5_NbO_3_ (B*mm*2 space group) (Figure 13C).

### 3.6. Piezoelectric Constant Measurement

**Figure 14:**
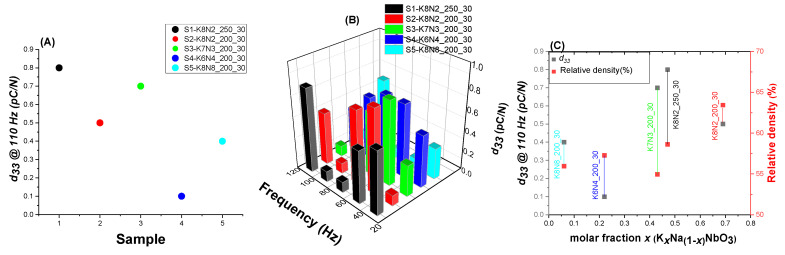
Piezoelectric constant *d*_33_ (**A**) for K8N2_250_30 (S 1), K8N2_200_30 (S 2), K7N3_200_30 (S 3), K6N4_200_30 (S 4) and K8N2_200_30 (S 5) at 110Hz; (**B**) d_33_ variation with frequency (30–110 Hz); (**C**) *d*_33_ variation with molar fraction *x* and the relative density of unsintered discs.

The piezoelectric coefficients have been unconventional, determined on unsintered discs of pressed KNN powders. Five samples which had the same dwelling time (30 min) and HTMW temperature of 200 or 250 °C were selected. In order to observe the influence of K*_x_*N_1−*x*_NbO_3_ composition on *d*_33_ values, the selected samples, K8N2_250_30 (S1), K8N2_200_30 (S2), K7N3_200_30 (S3), K6N4_200_30 (S4), K8N8_200_30 (S5), had different *x* values. As expected, it is observed that the highest *d*_33_ value is measured at 110 Hz for sample K8N2_250_30, which has an estimated formula K_0.47_Na_0.53_NbO_3_, followed by K7N3_200_30 with K_0.43_Na_0.57_NbO_3_, the closest two compositions to targeted solid solution (K_0.5_Na_0.5_NbO_3_) (Figure 14A and Table S8, supplementary data). The lowest *d*_33_ value stands for K6N4_200_30 at all frequencies tested, having K_0.22_Na_0.78_NbO_3_ composition (Figure 14B). Additionally, the piezoelectric constant depends on the ceramic morphostructure, with implication for material density after shaping and sintering, the highest relative density leading to the highest *d*_33_ value [68].

Without a proper ceramic densification by sintering, the holes, cracks and pores from the green samples’ micro-texture would concentrate the electric charges, stopping their movement to the surface, so the difference in measured electrical potential and *d*_33_ will be much lower. Therefore, the weak piezoelectric responses of all five compositions are caused by the very low densities obtained during discs preparation by pressing, compared to the theoretical density of KNN ceramics (4.51 g/cm^3^) [69]. As observed, the relative density measured did not exceed 65%, denoting a very low powder densification, in the absence of sintering treatment (Figure 14C). Additionally, the *d*_33_ measurement for KNN ceramics is usually performed after the polarization of the sintered cylindrical samples, condition not respected in this case, with negative implications for the direct piezoelectric effect.

## 4. Conclusions

Using the HTMW method and varying the K/Na ratio, the dwelling time and the temperature at high constant pressure (40 bar), fifteen specimens of K*_x_*N_1−*x*_NbO_3_ solid solution have been synthetized. It is observed that almost all specimens have both O and T crystal symmetry and K8N2_200_90 has, in addition, a 49% M phase. A slow increasing content of O phase could be observed for samples with (KOH + NaOH) = 10M, with increasing dwelling time and at the same time with a decrease in *x.*

The closest compositions to K_0.5_Na_0.5_NbO_3_ were found in three samples, K8N2, K7N3 and K8N8. A complete HTMW treatment at 250 °C for 30 min dwelling time and K/Na = 4/1 (K8N2) leads to a solid solution K*_x_*N_1−*x*_NbO_3_ with *x*~0.47, powder being characterized by a crystallinity of 63%, the coexistence of 67% O and 33% T crystal symmetry, average particle size of 221 nm and the highest piezoelectric effect *d*_33_ = 0.8 pC/N at 110 Hz. The second winner comes from the K7N3 series at 200 °C and 30 min dwelling time, with *x*~0.43, powder that has a crystallinity of 58% and 52% O + 48% T phases, average particle size of 147.5nm and *d*_33_ = 0.7 pC/N at 110 Hz (K7N3_200_30). The sample K7N3_200_30 shows the best piezoelectric effect (*d*_33_ = 1.2 pC/N) at 50 Hz, compared to all five specimens analyzed. The sample K8N8_200_90 highlighted a crystallinity degree of 59%, 52% O + 48% T coexisting phases, particles mean size of 165 nm and a *d*_33_ = 0.4 pC/N (110 Hz).

The increasing HTMW duration leads to dissolution effect activation (Oswald ripening), the nucleation speed increasing over crystal growth process, preserving the average particle size under 500 nm. The XRF spectra findings concerning the estimate composition of K*_x_*N_1−*x*_NbO_3_ were confirmed by Raman spectra and XRD plots, all results offer reliable explanations for SEM results and piezoelectric response.

Such powders are compositionally and structurally prepared to induce the best piezoelectric response used as a raw material for the creation of new biocomposites or for high-performance piezoelectric lead-free ceramics.

## Figures and Tables

**Table 1:** Molar fraction *x* variation with temperature, HTMW dwelling time and concentration of the precursor alkaline mixture.

Sample Precursors K/Na Rate	*x* Value (K*_x_*Na_1−*x*_NbO_3_) Evaluated from XRF Analysis Temperature(°C)_ Time (Minutes)						*x* Value (K*_x_*Na_1−*x*_NbO_3_) Evaluated from EDS-SEM Analysis Temperature(°C)_ Time (Minutes)					
	200_30	200_60	200_90	250_30	250_60	250_90	200_30	200_60	200_90	250_30	250_60	250_90
K8N8 1:1 = 1	0.06	0.10	0.44	-	-	-	0.03–0.07	0.02–0.05	0.24–0.28	-	-	-
K7N3 7:3 = 2.33	0.43	0.40	0.31	-	-	-	0.31–0.36	0.30–0.32	0.10–0.28	-	-	-
K6N4 3:2 = 1.5	0.22	0.15	0.09	-	-	-	0.13–0.18	0.17–0.18	0.01–0.09	-	-	-
K8N2 4:1 = 4	0.69	0.63	0.09	0.47	0.66	0.68	0.60–0.61	0.35–0.48	0.10–0.12	0.40–0.42	0.40–0.5	0.55–0.6

## Data Availability

Not applicable.

## Supplementary Materials

The following supporting information can be downloaded at: https://www.mdpi.com/article/10.3390/ma15155410/s1, Table S1: Samples definition according to concentration and HTMW conditions. Table S2: The variation of K and Na content in K*_x_*Na_1−*x*_NbO_3_ depending on the duration, precursor K/Na molar ratio and temperature of hydrothermal treatment, valuated by X-ray Fluorescence Spectroscopy. Table S3: Unit cell characteristics and phases content after Rietveld structural refinement for synthetized powder from series K8N2 at 200 °C and 250 °C, for 30–90 min HTMW treatment. Table S4: Unit cell characteristics and phases content after Rietveld structural refinement for synthetized powder from series K7N3 and K6N4 at 200 °C, for 30–90 min HTMW treatment. Table S5: Unit cell characteristics and phases content after Rietveld structural refinement for synthetized powder from series K8N8 at 200 °C, for 30–90 min HTMW treatment. Table S6: Variation of Goldschmidt tolerance factor (*t*) up to molar fraction *x* in K_x_Na_1−*x*_NbO_3_. Table S7: Average, minimum and maximum particle sizes from SEM images comparing with KNNss compositions and average crystallite sizes. Table S8: Piezoelectric coefficient *d*_33_ variation with the frequency and samples composition.
