# Supplementary material for: Alkali Niobate Powder Synthesis Using an Emerging Microwave-Assisted Hydrothermal Method
Source: Materials (Basel). 2022 Aug 6;15(15):5410. doi: 10.3390/ma15155410 (PMC9369762; doi:10.3390/ma15155410)
Supplement: Supplementary file 1 [file materials-15-05410-s001.zip › materials-1797717-supplementary.pdf]

Supplementary data

Table S1 Samples definition according to concentration and HTMW conditions

Crt. no.	Samples denomination	Alkaline solutions molarity	HTMW conditions
1	K8N8_200_30 K/Na=1/1	KOH+NaOH =8M+4M=16M	p= 40-47 bar, $T_{dwelling}$ = 200^{0}C $t_{dwelling}$=30 min
2	K8N8_200_60 K/Na=1/1	KOH+NaOH =8M+4M=16M	p= 40-47 bar, $T_{dwelling}$ = 200^{0}C $t_{dwelling}$=30 min
3	K8N8_200_90 K/Na=1/1	KOH+NaOH =8M+4M=16M	p= 40-47 bar, $T_{dwelling}$ = 200^{0}C $t_{dwelling}$=30 min
4	K6N4_200_30 K/Na=3/2	KOH+NaOH =6M+4M=10M	p= 40-47 bar, $T_{dwelling}$ = 200^{0}C $t_{dwelling}$=30 min
5	K6N4_200_60 K/Na=3/1	KOH+NaOH =6M+4M=10M	p= 40-47 bar, $T_{dwelling}$ = 200^{0}C $t_{dwelling}$=60 min
6	K6N4_200_90 K/Na=3/2	KOH+NaOH =6M+4M=10M	p= 40-47 bar, $T_{dwelling}$ = 200^{0}C $t_{dwelling}$=90 min
7	K7N3_200_30 K/Na=7/3	KOH+NaOH =7M+3M=10M	p= 40-47 bar, $T_{dwelling}$ = 200^{0}C $t_{dwelling}$=30 min
8	K7N3_200_60 K/Na=7/3	KOH+NaOH =7M+3M=10M	p= 40-47 bar, $T_{dwelling}$ = 200^{0}C $t_{dwelling}$=60 min
9	K7N3_200_90 K/Na=7/3	KOH+NaOH =7M+3M=10M	p= 40-47 bar, $T_{dwelling}$ = 200^{0}C $t_{dwelling}$=90 min
10	K8N2N_200_30 K/Na=4/1	KOH+NaOH =8M+2M=10M	p= 40-47 bar, $T_{dwelling}$ = 200^{0}C $t_{dwelling}$=30 min
11	K8N2N_200_60 K/Na=4/1	KOH+NaOH =8M+2M=10M	p= 40-47 bar, $T_{dwelling}$ = 200^{0}C $t_{dwelling}$=60 min
12	K8N2N_200_90 K/Na=4/1	KOH+NaOH =8M+2M=10M	p= 40-47 bar, $T_{dwelling}$ = 200^{0}C $t_{dwelling}$=90 min
13	K8N2N_250_30 K/Na=4/1	KOH+NaOH =8M+2M=10M	p= 40-70 bar, $T_{dwelling}$ = 250^{0}C $t_{dwelling}$=30 min
14	K8N2N_250_60 K/Na=4/1	KOH+NaOH =8M+2M=10M	p= 40-70 bar, $T_{dwelling}$ = 250^{0}C $t_{dwelling}$=60 min
15	K8N2N_250_90 K/Na=4/1	KOH+NaOH =8M+2M=10M	p= 40-70 bar, $T_{dwelling}$ = 250^{0}C $t_{dwelling}$=90 min

Table S2 The variation of K and Na content in $K_XNa_{1-X}NbO_3$ depending on the duration, precursor K/Na molar ratio and temperature of hydrothermal treatment, valuated by X-Ray Fluorescence Spectroscopy

Sample Precursor molar rate	XRF		% wt	Normalized molar %	XRF estimated formula for solid solution $K_XN_{1-X}NbO_3$ Molar ratio of Na and K in KNN
	Identified element	Estimated error			
K8N8_200_30 K: Na=1:1	Nb	0.41	67.35	100	$K_{0.06}Na_{0.94}NbO_3$
	K	0.16	9.66	93.51	
	Na	0.05	1.14	6.48	
K8N8_200_60 K: Na=1:1	Nb	0.18	85.84	100	$K_{0.10}Na_{0.90}NbO_3$
	Na	0.16	11.96	90.22	
	K	0.07	2.20	9.78	
K8N8_200_90	Nb	0.21	77.70	100	$K_{0.44}Na_{0.56}NbO_3$

K:Na=1:1	K	0.17	9.45	55.57	
	Na	0.15	12.85	44.43	
K6N4_200_30<br>K: Na=3:2	Nb	0.44	86.10	100	$K_{0.22}Na_{0.78}NbO_3$
	Na	0.15	9.32	77.61	
	K	0.1	4.57	22.39	
K6N4_200_60<br>K: Na=3:2	Nb	0.18	86.30	100	$K_{0.15}Na_{0.85}NbO_3$
	Na	0.15	10.48	84.67	
	K	0.09	3.23	15.33	
K6N4_200_90<br>K: Na=3:2	Nb	0.18	86.31	100	$K_{0.09}Na_{0.91}NbO_3$
	Na	0.16	11.64	90.61	
	K	0.07	2.05	9.39	
K7N3_200_30<br>K: Na=7:3	Nb	0.43	80.86	100	$K_{0.43}Na_{0.57}NbO_3$
	Na	0.14	8.40	57.08	
	K	0.16	10.74	42.92	
K7N3_200_60<br>K: Na=7:3	Nb	0.20	81.68	100	$K_{0.40}Na_{0.60}NbO_3$
	Na	0.15	8.51	59.60	
	K	0.14	9.81	40.40	
K7N3_200_90<br>K: Na=7:3	Nb	0.20	81.50	100	$K_{0.31}Na_{0.69}NbO_3$
	Na	0.15	10.40	68.59	
	K	0.14	8.10	31.41	
K8N2_200_30<br>K: Na=4:1	Nb	0.36	69.97	100	$K_{0.69}Na_{0.31}NbO_3$
	Na	0.21	6.32	31.19	
	K	0.11	23.71	68.81	
K8N2_200_60<br>K: Na=4:1	Nb	0.22	72.99	100	$K_{0.63}Na_{0.37}NbO_3$
	Na	0.20	6.91	36.88	
	K	0.13	20.10	63.12	
K8N2_200_90<br>K: Na=4:1	Nb	0.18	85.75	100	$K_{0.09}Na_{0.91}NbO_3$
	Na	0.16	12.21	91.05	
	K	0.07	2.04	8.95	
K8N2_250_30<br>K: Na=4:1	Nb	0.42	69.16	100	$K_{0.47}Na_{0.53}NbO_3$
	K	0.16	12.11	46.75	
	Na	0.12	8.11	53.24	
K8N2_250_60<br>K:Na=4:1	Nb	0.42	71.05	100	$K_{0.66}Na_{0.34}NbO_3$
	K	0.21	17.65	65.77	
	Na	0.17	5.4	34.22	

Table S3 Unit cell characteristics and phases content after Rietveld structural refinement for synthetized powder from series K8N2 at 200^{0}C and 250^{0}C, for 30-90 minutes HTMW treatment

Sample	K8N2_200_30		K8N2_200_60		K8N2_200_90			K8N2_250_30		K8N2_250_60		K8N2_250_90
KOH/NaOH (mol)	4:1		4:1		4:1			4:1		4:1		4:1
Phase ortorombic(O)/ tetragonal(T)/ monoclinic(M)	(O) 50.1%	(T) 49.9%	(O) 42.3%	(T) 57.7%	(O) 18.7%	(T) 32.5%	(M) 48.8%	(O) 67.4%	(T) 32.6%	(O) 55.9%	(T) 44.1%	(O) 100%
a (Å) (deviation)	5.6354 (0.1023)	3.9928 (0.000504)	5.6385 (0.00227)	4.01162 (0.00050)	5.6578 (0.00141)	3.9629 (0.00082)	5.5300 (0.00044)	5.6590 (0.00087)	3.9344 (0.00037)	5.6652 (0.0149)	3.9284 (0.00041)	5.6649 (0.00113)3
b(Å) (deviation)	3.9539 (0.008146)	3.9928 (0.000504)	3.9126 (0.00086)	4.01162 (0.00050)	3.9767 (0.000859)	3.9629 (0.00082)	7.7800 (0.00052)	3.9618 (0.00037)	3.9344 (0.00037)	3.9630 (0.00066)	3.9284 (0.00041)	3.9618 (0.00036)
c(Å) (deviation)	5.6375 (0.10469)	4.0287 (0.00073)	5.5390 (0.00150)	3.9728 (0.00071)	5.6875 (0.0014)	4.0309 (0.00098)	5.5728 (0.00056)	5.6783 (0.00091)	3.8944 (0.00043)	5.6629 (0.01470)	3.8917 (0.00045)	5.6768 (0.00112)
V(Å^3)	125.6163	64.2288	122.1988	63.9355	127.969	63.3053	239.7649	127.31	60.2892	127.1429	60.1031	127.4066

Crystallinity(%)		52.38		55.06		59.59		63.22		59.5		57.4%	
Average crystallite size(nm)		20.3687	6.07816	13.8890	14.9435	24.78025	14.3814	33.9802	19.7579	25.3949	16.7811	28.7533	20.3680
Standard deviation(nm)		6.6195	1.3643	4.8898	5.4448	8.1511	5.2644	18.0704	7.7729	17.7106	4.6056	17.6814	4.8422
Micro-strain (%)		1.4476	0.4498	0.6533	0.6193	0.3565	0.6473	0.2690	0.4642	0.4066	0.5301	0.3463	0.4345
Standard deviation(%)		0.2149	0.0488	0.0379	0.0681	0.0268	0.0374	0.0291	0.03319	0.05414	0.0556	0.0384	0.0600
Agreed indices	R_{exp}	3.9025		3.9903		3.8206		4.2508		4.2792		3.8504	
	R_p	4.5754		4.0156		3.5724		3.9194		4.3810		4.6338	
	wR_p	6.1994		5.2314		4.6668		5.4001		6.1591		6.711	
	GOF	2.52		1.71		1.49		1.6139		2.07		3.037	

Table S4 Unit cell characteristics and phases content after Rietveld structural refinement for synthetized powder from series K7N3 and K6N4 at 200^{0}C , for 30-90 minutes HTMW treatment

Sample		**K7N3_200_30**		**K7N3_200_60**		**K7N3_200_90**		**K6N4_200_30**		**K6N4_200_60**		**K6N4_200_90**	
KOH/NaOH (mol)		7:3						3:2					
Phase ortorombic(O)/ tetragonal(T)/ monoclinic(M)		(O) 52.1%	(T) 47.9%	(O) 52.2%	(T) 47.8%	(O) 62.2%	(T) 37.8%	(O) 75.4%	(T) 24.6%	(O) 77.1%	(T) 22.9%	(O) 56.4%	(T) 43.6%
a (Å) (deviation)		3.8938 (0.000433)	3.9707 (0.000519)	3.8922 (0.00042)	3.9671 (0.00058)	3.8881 (0.00029)	3.9587 (0.00061)	3.8891 (0.00030)	3.9427 (0.00243)	3.8888 (0.00027)	3.9284 (0.00041)	3.8856 (0.00023)	3.9054 (0.00035)
b(Å) (deviation)		5.5755 (0.0010)	3.9707 (0.000519)	5.5314 (0.00071)	3.9671 (0.00058)	5.5243 (0.00050)	3.9587 (0.00061)	5.5266 (0.00050)	3.9427 (0.00243)	5.5242 (0.00046)	3.9284 (0.00041)	5.5213 (0.00040)	3.9054 (0.00035)
c(Å) (deviation)		5.5349 (0.00075)	4.0186 (0.00068)	5.5743 (0.00093)	4.0213 (0.00076)	5.5701 (0.00058)	4.0155 (0.00075)	5.5703 (0.00055)	4.0043 (0.00163)	5.5697 (0.00050)	3.8917 (0.00045)	5.5738 (0.00041)	3.9403 (0.00069)
V(Å^3)		120.1669	63.3593	120.0152	63.2876	119.6425	62.9363	119.7261	62.2482	119.6524	60.1031	119.6014	60.1017
Crystallinity(%)		57.73		64.35		53.19		68.31		63.48%		49.42%	
Average crystallite size(nm)		25.7422	14.5243	24.1923	12.3563	32.7784	12.5505	32.7972	9.3316	34.9922	8.5526	52.6506	19.7881
Standard deviation(nm)		11.0275	5.4483	12.8279	4.8849	12.7393	5.5553	13.2842	5.9515	15.3766	5.1954	34.6371	11.7841
Micro-strain (%)		0.3570	0.6305	0.4345	0.7574	0.2782	0.7504	0.2789	1.0651	0.2634	1.1456	0.1841	0.5015
Standard deviation(%)		0.0320	0.0356	0.2587	0.0268	0.0324	0.0528	0.0262	0.1168	0.0252	0.1278	0.0254	0.0836
Agreed indices	**R_{exp}**	3.8985		4.2594		4.2779		4.1856		3.7852		4.2435	
	**R_p**	4.2040		4.0705		4.2917		4.6026		4.5123		4.5468	
	**wR_p**	5.5669		5.5020		5.9366		6.2738		6.1138		6.1852	
	**GOF**	2.03		1.66		1.92		2.24		2.6		2.12	

Table S5 Unit cell characteristics and phases content after Rietveld structural refinement for synthetized powder from series K8N8 at 200^{0}C, for 30-90 minutes HTMW treatment

Sample	K8N8_200_30	K8N8_200_60		K8N8_200_90	
KOH/NaOH (mol)	1:1				
Phase ortorombic(O)/ tetragonal(T)/ monoclinic(M)	(O) 100%	(O) 52.6%	(T) 47.4%	(O) 51.5%	(T) 48.5%
a (Å) (deviation)	3.8873 (0.00014)	3.8880 (0.00030)	3.9059 (0.00030)	3.8866 (0.00027)	3.9075 (0.00032)
b(Å) (deviation)	5.5129 (0.00027)	5.5237 (0.00053)	3.9059 (0.00030)	5.2185 (0.00049)	3.9075 (0.00032)
c(Å) (deviation)	5.5675 (0.00024)	5.5749 (0.00056)	3.9492 (0.00051)	5.5726 (0.00051)	3.9475 (0.00064)

V(Å³)		119.3173	119.7289	60.2531	119.5964	60.2737
Crystallinity(%)		56.25	61		58.79	
Average crystallite size(nm)		47.2716	42.7854	26.5016	48.6622	20.9108
Standard deviation(nm)		20.7640	22.6923	13.7188	28.5742	10.6683
Micro-strain (%)		0.1400	0.2199	0.3651	0.1956	0.4615
Standard deviation(%)		0.0235	0.02490	0.03863	0.0253	0.0478
Agreed indices	R_{exp}	4.0219	4.1307		4.0987	
	R_p	5.7310	4.3981		4.5190	
	wR_p	8.003	5.9732		6.1623	
	GOF	3.95	2.09		2.26	

Table S6 Variation of Goldschmidt tolerance factor (t) up to molar fraction x in $K_xNa_{1-x}NbO_3$

x from $K_xNa_{1-x}NbO_3$	(1-x) from $K_xNa_{1-x}NbO_3$	R_{K^+}(Å) (CN=12)	R_{Na^+} (Å) (CN=12)	$R_{Nb^{5+}}$ (Å) (CN=6)	$R_{O^{2-}}$ (Å) (CN=6)	Goldschmidt tolerance factor t (equation 3)	$K_xNa_{1-x}NbO_3$
0	1	1.64	1.39	0.64	1.4	0.964558	$NaNbO_3$
1	0					1.050373	$KNbO_3$
0.5	0.5					1.007465	$K_{0.5}Na_{0.5}NbO_3$
0.06	0.94					0.969707	$K_{0.06}Na_{0.94}NbO_3$
0.09	0.91					0.972281	$K_{0.09}Na_{0.91}NbO_3$
0.1	0.9					0.973139	$K_{0.1}Na_{0.9}NbO_3$
0.15	0.85					0.97743	$K_{0.15}Na_{0.85}NbO_3$
0.22	0.78					0.983437	$K_{0.22}Na_{0.78}NbO_3$
0.31	0.69					0.99116	$K_{0.31}Na_{0.69}NbO_3$
0.4	0.6					0.998884	$K_{0.4}Na_{0.6}NbO_3$
0.43	0.57					1.001458	$K_{0.43}Na_{0.57}NbO_3$
0.44	0.56					1.002316	$K_{0.44}Na_{0.56}NbO_3$
0.47	0.53					1.004891	$K_{0.47}Na_{0.53}NbO_3$
0.63	0.37					1.018621	$K_{0.63}Na_{0.37}NbO_3$
0.66	0.34					1.021196	$K_{0.66}Na_{0.34}NbO_3$

Table S7 Average, minimum and maximum particle sizes from SEM images comparing with KNNss compositions and average crystallite sizes

Sample	XRF estimated formula of $K_XN_{1-X}NbO_3$	Average particle size (nm)	Standard Deviation (nm)	Minimum particle size(nm)	Maximum particle size(nm)	Average crystallite size from Rietveld refinement (O+T) (nm)
K8N8N_200_30	$K_{0.06}N_{0.94}NbO_3$	224.7	12.0650	1.54	754	47.27005
K8N8N_200_60	$K_{0.10}N_{0.90}NbO_3$	179.7	13.3992	0.28	583	35.06328
K8N8N_200_90	$K_{0.44}N_{0.56}NbO_3$	164.7	13.1887	0.287	676.5	

						35.20125
K6N4N_200_30<br>K6N4N_200_60<br>K6N4N_200_90	$K_{0.22}N_{0.78}NbO_3$<br>$K_{0.15}N_{0.85}NbO_3$<br>$K_{0.09}N_{0.91}NbO_3$	317.8<br>479.5<br>274.6	9.6218<br>9.6218<br>9.3580	0.59<br>26.8<br>85.4	972.7<br>980.8<br>989.5	27.01884<br>28.93524<br>38.31868
K7N3N_200_30<br>K7N3N_200_60<br>K7N3N_200_90	$K_{0.43}N_{0.57}NbO_3$<br>$K_{0.40}N_{0.60}NbO_3$<br>$K_{0.31}N_{0.69}NbO_3$	147.5<br>98.5<br>403	11.2016<br>11.3270<br>14.5447	0.398<br>0.56<br>39.1	971<br>593.1<br>1209	20.36562<br>18.53048<br>25.12684
K8N2N_200_30<br>K8N2N_200_60<br>K8N2N_200_90	$K_{0.69}N_{0.31}NbO_3$<br>$K_{0.63}N_{0.37}NbO_3$<br>$K_{0.09}N_{0.91}NbO_3$	463.6<br>180.9<br>116.5	11.7889<br>10.7421<br>12.2353	35.8<br>24.3<br>0.338	1593<br>577.9<br>583.3	13.20871<br>14.49162<br>25.8896
K8N2N_250_30<br>K8N2N_250_60<br>K8N2N_250_90	$K_{0.47}N_{0.53}NbO_3$<br>$K_{0.66}N_{0.34}NbO_3$<br>$K_{0.68}N_{0.32}NbO_3$	221.3<br>369.4<br>301	11.2744<br>19.6301<br>30.29	64<br>0.49<br>66	775<br>2950<br>1507	21.58<br>22.06<br>20.36

Table S8 Piezoelectric coefficient d_{33} variation with the frequency and samples composition

Frequency	S1 - K8N2-250-30	S 2 - K8N2-200-30	S 3 - K7N3-200-30	S 4 - K6N4-200-30	S 5 - K8N8-200-30
(Hz)	(pC/N)	(pC/N)	(pC/N)	(pC/N)	(pC/N)
	d_{33}	d_{33}	d_{33}	d_{33}	d_{33}
30	0.6	0.1	0.9	0.3	0.5
50	0.5	0.8	1.2	0.8	0.7
70	0.1	0.7	0.6	0.6	0.7
90	0.1	0.1	0.7	0.2	0.6
110	0.8	0.5	0.7	0.1	0.4
